# A United Response to COVID-19—an Artist’s Perspective

**DOI:** 10.3201/eid2813.AC2813

**Published:** 2022-12

**Authors:** Byron Breedlove, Cynthia H. Cassell, Pratima L. Raghunathan

**Affiliations:** Centers for Disease Control and Prevention, Atlanta, Georgia, USA

**Keywords:** COVID-19, art science connection, emerging infectious diseases, art and medicine, about the cover, global health, epidemiologic surveillance, emergency preparedness and response, immunization and clinical services, A United Response to COVID-19, an Artist’s Perspective, coronavirus disease, respiratory infections, severe acute respiratory syndrome coronavirus 2, SARS-CoV-2, coronaviruses, virus, Vasundhara Tolia, the world united

**Figure:**
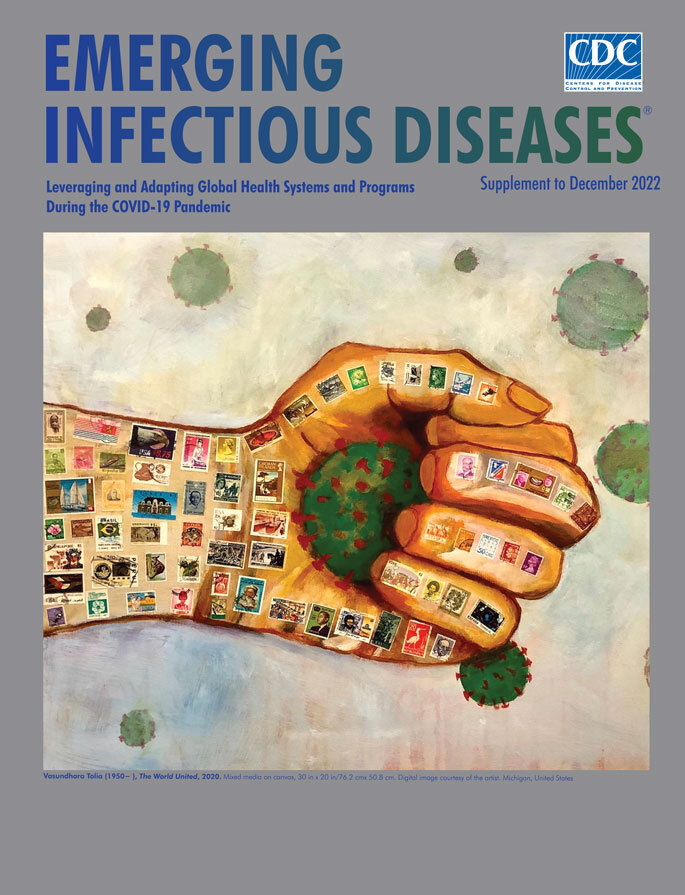
**Vasundhara Tolia (1950−), *The World United*, 2020.** Mixed media on canvas, 30 in x 20 in/76.2 cm x 50.8 cm. Digital image courtesy of the artist. Michigan, United States.

During mid-March 2020, the World Health Organization (WHO) declared that the spread of COVID-19, the respiratory illness caused by SARS-CoV-2, was a pandemic. This novel emerging infectious disease spread insidiously and swiftly around the globe, undeterred by geographic borders. Countries reacted to COVID-19 with attempts to control transmission, including isolation and quarantine orders, social distancing recommendations, and mask requirements. Responses at the local, national, regional, and international levels involved public health experts, field epidemiologists (disease detectives), clinicians, researchers, policy makers, political leaders, and civil authorities. 

Artists from across the globe also responded to the effects of COVID-19 in myriad ways, communicating a wide range of perspectives and experiences about the pandemic through imagery, music, dance, and writing. Efforts to collect and share some of this artistic output via online platforms helped connect artists and audiences to a greater degree than would otherwise have been possible during the pandemic. For example, in spring 2020 the *Washington Post* invited readers to submit artwork created during the early months of the COVID-19 outbreak. The paper featured 20 works, selected from more than 650 submissions, in the article “The Best Art Created by *Washington Post* Readers during the Pandemic.” Michael Cavna, a writer-artist-cartoonist who penned the story, explained, “The Post considered not only the quality and creativity of the art, but also the fascinating accompanying backstories. Enduring quarantines, some artists rendered what isolation and loneliness felt like, while others depicted longed-for social scenes from a pre-pandemic time.” 

*The World United,* the cover art for this special supplement issue of *Emerging Infectious Diseases*, was among those finalists. Vasundhara Tolia, who created this image, took a somewhat different approach from other artists. Tolia, originally from India, is now a retired pediatric gastroenterologist and served as a tenured professor of pediatrics and as a consultant and attending physician at hospitals in the Detroit, Michigan, USA, area, where she currently lives. She has embarked on a second career as an artist, and her work has been shown in group exhibitions in several states, many online national exhibitions, and several solo shows. In that same *Washington Post* article, Tolia wrote about her painting, “Medicine has always been my first passion. And during these unprecedented, tumultuous times, it beckons me again as I watch helplessly from the sidelines now. Since my retirement as a physician, I’ve poured my creativity into art and poetry, so creating this kind of response came naturally to me.” 

Tolia recounted her inspiration for creating *The World United* in more detail. “The world did seem to have come together in response to dealing with the pandemic. In some ways we were cohesive, especially with the creation of the vaccine. To show this togetherness, I wanted to show the world combating this virus. I wanted to depict something new and different, so I made this hand gripping the virus although this elusive particle still escaped because of its invasive properties and ubiquitous presence. Rather than a map of the world, I chose to use stamps from as many countries of the world that I could fit on this hand. My sons used to collect stamps when young, so I looked in their collections and used some of them. That’s how this painting was conceived” (V. Tolia, pers. comm., email, 2022 May 8).

More than 60 different stamps, each one a miniature painting, are rendered with such attention to detail that cancellation marks are visible on many. The hand clutches one of the coronaviruses as the others float away. Tolia’s image, created during a time when the world was starting to come to grips with the pandemic, elicits a sense of esprit de corps reminiscent of the now famous “We Can Do It” posters Pittsburgh artist J. Howard Miller created to inspire American workers during World War II.

Since launching its response to the COVID-19 pandemic, the Centers for Disease Control and Prevention (CDC) has focused on learning about the disease how it spreads and how it affects people and communities in the United States and around the world. Drawing on CDC staff, funded programs, and partnerships with many countries, CDC’s global COVID-19 response has provided epidemiologic surveillance, laboratory support, emergency preparedness and response, and immunization and clinical service delivery resources to countries and vulnerable populations. 

The response exemplifies working collaboratively with global, national, and local public health leadership, including WHO, ministries of health, and community leaders. Those partnerships were strengthened at all levels as public health practitioners generated scientific knowledge, refined technical approaches to prevent and mitigate COVID-19, and identified areas for continued improvement and reassessment. 

In an interview with writer and artist Linda Sienkiewicz, Tolia discussed her views on art: “All the ways we separate ourselves, be it by our age, ethnicity, culture, geography and even interests; all of these boundaries melt away when we take in and connect with art and creative forms of expression.” Art and science can connect people regardless of place or origin. During the pandemic, people turned to art and science to understand and make sense of the world, and both disciplines remind us that unity is possible.

## References

[1] Cassell CH, Raghunathan PL, Henao O, Pappas-DeLuca KA, Rémy WL, Dokubo EK. Overview from CDC and its partners on global responses to the COVID-19 pandemic. Emerg Infect Dis. 2022;28: S4-7. 10.3201/eid2813.221733 36502408 PMC9745249

[2] Cavna M. The best art created by Washington Post readers during the pandemic [cited 2022 May 5]. The Washington Post. https://www.washingtonpost.com/arts-entertainment/2020/07/06/art-pandemic-readers

[3] Centers for Disease Control and Prevention. Center for Global Health [cited 2022 Aug 3]. https://www.cdc.gov/globalhealth/covid-19/index.html

[4] Centers for Disease Control and Prevention. COVID-19. CDC’s response [cited 2022 Aug 18]. https://www.cdc.gov/coronavirus/2019-ncov/cdcresponse/index.html

[5] Sienkiewicz L. What, why, how: Vasundhara Tolia [cited 2022 Jun 23]. http://lindaksienkiewicz.com/what-why-how-vasundhara-tolia

[6] Tolia V. Fine art. About the artist [cited 2022 May 5]. https://www.vasutolia.com/know-more-about-the-artist-vasu-tolia.html

